# Core principles of evolutionary medicine: A Delphi study

**DOI:** 10.1093/emph/eox025

**Published:** 2017-12-26

**Authors:** Daniel Z Grunspan, Randolph M Nesse, M Elizabeth Barnes, Sara E Brownell

**Affiliations:** 1 Center for Evolution and Medicine, Arizona State University, Tempe, AZ 85287, USA; 2 School of Life Sciences, Arizona State University, Tempe, AZ 85287, USA

**Keywords:** core principles, evolutionary medicine, education, Delphi study, core concepts

## Abstract

**Background and objectives:**

Evolutionary medicine is a rapidly growing field that uses the principles of evolutionary biology to better understand, prevent and treat disease, and that uses studies of disease to advance basic knowledge in evolutionary biology. Over-arching principles of evolutionary medicine have been described in publications, but our study is the first to systematically elicit core principles from a diverse panel of experts in evolutionary medicine. These principles should be useful to advance recent recommendations made by The Association of American Medical Colleges and the Howard Hughes Medical Institute to make evolutionary thinking a core competency for pre-medical education.

**Methodology:**

The Delphi method was used to elicit and validate a list of core principles for evolutionary medicine. The study included four surveys administered in sequence to 56 expert panelists. The initial open-ended survey created a list of possible core principles; the three subsequent surveys winnowed the list and assessed the accuracy and importance of each principle.

**Results:**

Fourteen core principles elicited at least 80% of the panelists to agree or strongly agree that they were important core principles for evolutionary medicine. These principles over-lapped with concepts discussed in other articles discussing key concepts in evolutionary medicine.

**Conclusions and implications:**

This set of core principles will be helpful for researchers and instructors in evolutionary medicine. We recommend that evolutionary medicine instructors use the list of core principles to construct learning goals. Evolutionary medicine is a young field, so this list of core principles will likely change as the field develops further.

## Teaser

This study analyzed survey responses from 46 experts to create a consensus list of 14 Core Principles for Evolutionary Medicine. These principles illustrate the wide range of evolutionary applications in medicine, including seeking evolutionary explanations for vulnerability to disease. Focusing instruction on these principles will advance evolutionary understanding in medicine.

## INTRODUCTION

Evolution is recognized as a core concept in biology [1,2] and has been described as essential for making sense of everything in biology [3], but historically it has not been emphasized in physician training [4–6]. The recent recommendations from the Association of American Medical Colleges and the Howard Hughes Medical Institute listed evolutionary thinking as a core competency for pre-medical education [7] and the medical college admissions test (MCAT) was recently changed to include evolution. However, despite these changes and continued advances in evolutionary applications to health and medicine [8], evolutionary biology remains absent from most medical curricula [4].

Evolutionary medicine, the field that applies the principles of evolutionary biology to health and disease, is a nascent, but growing response to this challenge of integrating evolution with medicine. Sometimes referred to as Darwinian Medicine or Evolution and Medicine, it has grown exponentially since the early 1990s [8], contributing to a greater understanding of topics paramount to human health including aging [9,10], reproductive health [11,12], immune function [13,14], infectious disease [15,16], cancer [17,18], behavioral disorders and mental health [19,20], microbiomes [21], veterinary medicine [22], inflammation [23] and diet [24]. An evolutionary perspective has not only proven to be a powerful and broad lens for medical advancement, but can also be used as a comprehensive scaffold for organizing medical knowledge that otherwise remains unconnected [25].

A course in evolutionary medicine can increase the relevance of evolutionary theory to students, enrich biological understanding of disease, and provide a unique perspective on how evolution can affect human health and disease. Although there is a need to develop evolutionary medicine courses, a current impediment is the lack of consensus on core principles that unite the field. Core principles are ideas that are central to a field and broad in their explanatory power. Ideally, a full set of core principles covers the breadth of work in a field while minimizing redundancy between principles. Mastering all principles in depth should qualify an individual as meeting minimum proficiency in a field.

Identifying a field’s core principles can help guide instructors as to what to teach [1]. They can also provide a common scaffolding that minimizes the danger of courses being built around a series of disconnected facts that leave students without an adequate understanding of the field as a whole and without the skills needed to transfer important concepts to other areas of biology [26]. Indeed, research suggests that student retention and understanding are increased if fewer large ideas are taught in greater depth, as opposed to teaching a greater breadth of content at a shallower level [27,28]. Thus, while the abundance of interesting facts and ideas relevant to evolution and medicine often makes it easy to keep students engaged, it can also be over-whelming and distract from the most important take-home messages in evolutionary medicine curricula. By organizing vast amounts of information into a smaller network of key ideas, a curricular focus on core principles can help students to understand the significance and interrelationships among bits of information. Further, focusing learning goals on core principles, which often represent more abstract ideas as opposed to facts, can help move instruction away from lower level cognitive goals such as rote memorization [25]. The American Association for the Advancement of Science’s (AAAS) Vision and Change report recommends pedagogy that teaches students to think through the big ideas of scientific fields, rather than teaching isolated facts [1]. Meeting these recommendations requires having some consensus on a set of core principles for a field.

Members of the evolutionary medicine community have not yet attempted to systematically define the evolutionary principles that are essential for students to master. However, a growing number of articles provide recommendations. Nesse et al. [29] constructed a learning goal framework for pre-medical and medical training based on the AAMC-HHMI report’s broad call for the inclusion of evolutionary biology into medical training [7]. Their recommendations stemmed from years of discussions among diverse experts in evolutionary medicine. Building off of the recommendations from Nesse et al. [29], Graves et al. [25] further outlined how evolutionary concepts are important for medical education, including the importance of using Bloom’s Taxonomy to ensure deep level understanding of evolutionary concepts [30]. Nesse and Schiffman [5], and later Hidaka et al. [4], surveyed medical school deans about the inclusion of evolutionary principles in their curriculum. While the reported importance and coverage of evolutionary principles in medical school curriculum increased over the 12 years, half of the deans in the 2015 study anticipated that including evolution in the curriculum would cause controversy. These hesitations by medical education leaders suggest that it is better to introduce evolutionary medicine in under-graduate curricula. Motivated to help increase the integration of human health into evolution education, Antolin et al. [31] provided biomedical examples that exemplify evolutionary concepts that can be integrated into classrooms. Other articles have provided descriptive accounts of courses in evolution and medicine as guides to help others design their own courses [32,33] and several textbooks have recently been published on evolutionary medicine [34–36]. This prior work provides a platform that helps to initiate our attempt to create a consensus view of core principles for the field of evolutionary medicine.

## METHODOLOGY

### IRB

Our institutional review board (#00005090) approved this study.

### The Delphi method

The current study used the Delphi method to generate and validate a list of core principles. The Delphi method utilizes expert opinions to address questions where answers are somewhat subjective in nature, and thus lack traditional analytical solutions. Many kinds of questions have been addressed through the Delphi method, including educational questions surrounding curricular design [37–39] and specifically the identification of disciplinary core principles. Examples of other fields that have used this method to identify core principles include clinical pharmacology and therapeutics [40], family medicine [41] and biomedical laboratory science [42].

The Delphi method starts with an initial question and uses feedback from an expert panel to gain resolution. This method employs a specific communication structure where panelists do not directly communicate with one another, but instead send responses to the research team. This structure allows consensus seeking to utilize a variety of opinions, while excluding the distorting influence of dissent or agreement that may occur due to social pressures, such as influence from individuals who may have higher status [43,44].

The Delphi method often starts with a survey requesting open-ended responses from panelists about the topic of interest. Upon receiving and analyzing responses to this initial survey, the research team sends a summary back to each panelist along with a second survey asking panelists to evaluate responses from the first survey. This process is performed iteratively in additional surveys until there is either consensus agreement amongst the panel, or a lack of consensus that identifies the reasons for disagreements. The current study followed this general format using four surveys to elicit core principles in evolutionary medicine.

### Panel selection

Panel selection is a critical component of a Delphi study. Panelists must represent diverse opinions from qualified experts. Evolutionary medicine is a transdisciplinary field, with contributions from biologists, anthropologists, and human and animal medical professionals, among others. Because the validity of Delphi results relies on having diverse experts on the panel, panel construction was performed with the transdisciplinary nature of evolutionary medicine in mind. To begin identifying panelists, six recognized experts in the field were invited via e-mail to each nominate individuals to participate in the Delphi panel. This invitation specifically asked these experts to identify other individuals who have a good grasp of the field, including both individuals who have a broad view and those whose perspective is more specialized. In total, five experts responded to this e-mail and made 35 total nominations with 24 unique individuals listed. Because a larger panel specifically constructed to achieve diversity in expertise and background was desired, the research team then identified 32 more individuals based on their active participation in the EvMed community through publications and participation in the International Society for Evolution, Medicine and Public Health conference (ISEMPH). All six of the initial experts were also included on this panel, either through being nominated initially by one of the other experts or through their inclusion in the list of 32 additional panelists. This process aimed to identify panelists who would provide perspectives that were diverse by fields, geographic locations and genders. Members of the same institution of the team that conducted the survey were not included in the panel to minimize bias because one of the study authors was their supervisor, to avoid over-representation of local points of view, and because local conversations and relationships at the university could erode the benefits of the anonymous nature of the Delphi structure. In total, 56 panelists were identified and invited to participate in the Delphi study.

The final panel consisted of 19 females and 37 males. At the time of the study, 40 panelists worked in North America, 12 in Europe and four in other continents. While we did not collect data on the age of our panelists, we can look at the number of years since their first publication to understand the academic age of our panel. The average number of years since the first publication of panelists was 26.3, with a standard deviation of 10.5, indicating a more senior panel based on the time they have been publishing. The breadth of expertise in the panel is hard to define, as many (if not all) panelists have diverse areas of expertise, and are hard to categorize. We classified panelists as primarily biologists (*n* = 26), anthropologists (*n* = 11), medical doctors (*n* = 12), or into a catch-all ‘other’ category of researchers or professionals in other fields (*n* = 7). A summary of these classifications, along with a co-authorship network of panelists, display the heterogeneity in expertise and publication histories (Supplementary Material).

### Over-view of survey process

Four sequential online surveys were administered to all 56 panelists. All four surveys included a statement about consent, the purpose of the survey and three definitions of ‘core principle’ (Box 1) following methods from a previous study with similar goals [45]. The first survey asked the panelists to ‘list the big ideas that you feel are important in the field of Evolutionary Medicine’ and to ‘elaborate on what the idea is, and why you think it is important’.

Two members of the research team (DZG and MEB) independently read all responses to the first survey and compiled a list of emergent core principles. These two lists were very similar, with few areas of disagreement, mainly about the scope of different principles; some principles could be subsumed within larger ones. Disagreements about the identification and hierarchy of distinct principles and their wording were resolved through discussion and further evaluation of panelist responses by the entire research team (DZG, RMN, MEB and SEB). To help avoid redundancy among core principles, a decision was made to divide the principles into ‘core’ principles that tended to be broad, and ‘sub’ principles that tended to be more specific. Panelists evaluated these core principles and sub-principles in the second survey.

Panelists received individual emails inviting participation in the second survey, which provided a list of all ‘core’ and ‘sub’ principles from the first round of the survey, with potential over-laps in core and sub-principles highlighted. Panelists were asked to rate the principles based on their importance to evolutionary medicine, and were given the option to comment on each principle. These comments could include why they rated a principle as they did, thoughts about specific wording, or about the initial categorization of principles as a core or a sub-principle. Responses to this survey were independently analyzed by two members of the research team (DZG and MEB) and further discussed among the full research team (DZG, MEB, RMN and SEB), as the basis for refining an updated list of core principles. The subsequent two surveys no longer included the sub principles from round two, and asked panelists to rate and comment only on the updated lists of potential core principles. At each stage, the research team modified principles when several panelists made similar comments, or if a single comment illuminated inaccuracies or obscurity in the wording of a principle. Table 1 provides an over-view of each survey, including the purpose, the response rate, along with the number of evaluated principles at each stage. A full over-view of the methods and survey results are provided in the Supplementary Material.

**Table 1.** Overview of the four Delphi surveys including their purpose, types of participant response, as well as the number of responses out of the 56 panelists each survey was sent to

	Purpose of survey	Response	Number of responses
Round 1	Generate initial list of core principles.	Open ended response.	30
Round 2	Rate importance of core principles; Rate importance of sub-principles.	Likert and open-ended response.	37
Round 3	Rate accuracy and importance of core principles. Sub-principles not rated in survey.	Likert and open-ended response.	30
Round 4	Rate accuracy and importance of core principles. Sub-principles not rated in survey.	Likert and open-ended response.	28

## RESULTS

**Figure 1.**
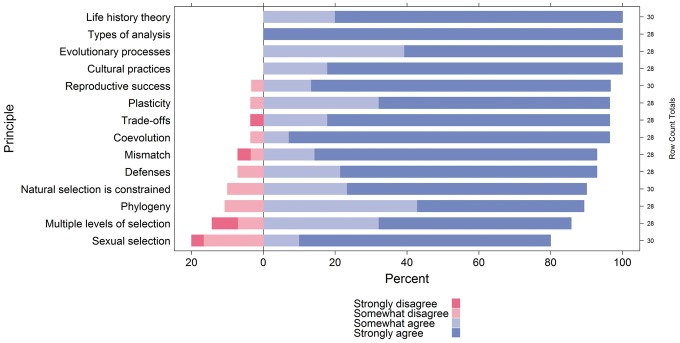
Importance rankings for the core principle that achieved consensus in the third or fourth survey

Fourteen core principles were endorsed by at least 80% of the final panel at the level of somewhat or strongly agreeing with its importance, a level of agreement previously recommended as a mark for consensus [46] (Table 2). Panelists rated the longer statements (e.g. ‘Both proximate (mechanistic) and ultimate (evolutionary) explanations are needed …’), while the research team created the short names for each principle (e.g. ‘Types of Analysis’). The research team grouped these principles based on how similar they were to one another after the completion of the study to help organize the principles based on similarity. These groups included: (i) Question framing includes one principle about the different types of questions addressed in biology; (ii) Evolution I and Evolution II, which were general evolutionary principles, with the principles in Evolution II more complex than those in Evolution I; (iii) Evolutionary-Tradeoffs includes both Trade-offs and Life History Theory, which are closely related concepts as they apply to health; (iv) Reasons for vulnerability include the two principles that represent direct evolutionary explanations for disease and (v) Culture includes the one principle that discusses the impacts of cultural practices. Figure 1 displays Likert scale responses to the question: ‘This is an important core principle for Evolutionary Medicine’ for each principle.

**Table 2.** Core Principles of Evolutionary Medicine

Topic	Core principle
Types of explanation (question framing)	Both proximate (mechanistic) and ultimate (evolutionary) explanations are needed to provide a full biological understanding of traits, including those that increase vulnerability to disease.
Evolutionary processes (evolution I)	All evolutionary processes, including natural selection, genetic drift, mutation, migration and non-random mating, are important for understanding traits and disease.
Reproductive success (evolution I)	Natural selection maximizes reproductive success, sometimes at the expense of health and longevity.
Sexual selection (evolution I)	Sexual selection shapes traits that result in different health risks between sexes.
Constraints (evolution I)	Several constraints inhibit the capacity of natural selection to shape traits that are hypothetically optimal for health.
Trade-offs (evolutionary trade-offs)	Evolutionary changes in one trait that improve fitness can be linked to changes in other traits that decrease fitness.
LHT (evolutionary trade-offs)	Life history traits, such as age at first reproduction, reproductive lifespan and rate of senescence, are shaped by evolution, and have implications for health and disease.
Levels of selection (evolution II)	Vulnerabilities to disease can result when selection has opposing effects at different levels (e.g. genetic elements, cells, organisms, kin and other levels).
Phylogeny (evolution II)	Tracing phylogenetic relationships for species, populations, traits or pathogens can provide insights into health and disease.
Coevolution (evolution II)	Coevolution among species can influence health and disease (e.g. evolutionary arms races and mutualistic relationships such as those seen in the microbiome).
Plasticity (evolution II)	Environmental factors can shift developmental trajectories in ways that influence health and the plasticity of these trajectories can be the product of evolved adaptive mechanisms.
Defenses (reasons for vulnerability)	Many signs and symptoms of disease (e.g. fever) are useful defenses, which can be pathological if dysregulated.
Mismatch (reasons for vulnerability)	Disease risks can be altered for organisms living in environments that differ from those in which their ancestors evolved.
Cultural practices (culture)	Cultural practices can influence the evolution of humans and other species (including pathogens), in ways that can affect health and disease (e.g. anti-biotic use, birth practices, diet, etc.).

The full wording of each principle was approved by at least 80% of panelists after the fourth round of the Delphi survey. The research team labeled each principle with a topic name and grouped these principles based on their relation to one another after the completion of the study to help organize the principles. Descriptions of the groups are as follows: Question framing includes one principle about the different types of questions addressed in biology. Evolution I and Evolution II include general evolutionary principles, with the principles in Evolution II more complex than those in Evolution I. Evolutionary-Tradeoffs includes both Trade-offs and Life History Theory, which are closely related concepts as they apply to health. Reasons for vulnerability include the two principles that represent direct evolutionary explanations for disease. Culture includes the one principle that discusses the impacts of cultural practices.

The format of the final core principles condenses broad abstract ideas into a necessarily short and condensed form. Different opinions about optimal wording reduced agreement between panelists on the importance of several principles. While consensus for 14 core principles was reached, persistent confusion and disagreements arising from the wording suggested a need for elaboration on each. Below we expand on each principle to elaborate those meanings, and illustrate some of the common comments and issues of panelists.

*Types of analyses* (100% agreement)—Both proximate (mechanistic) and ultimate (evolutionary) explanations are needed to provide a full biological understanding of traits, including those that increase vulnerability to disease. Understanding evolutionary medicine requires understanding the kinds of questions asked in the field, especially the difference between proximate and evolutionary explanations. Tinbergen formulated a framework including four categories of explanations for traits [47–49]. Respondents had different opinions about whether this principle should include all four categories, whether Tinbergen’s name should be used in the principle, and whether the word ‘analyses’ or ‘questions’ should be used. Regardless of wording, this principle provides an essential foundation for recognizing the several complementary kinds of explanations that can be used across the life sciences.

*Evolutionary processes* (100% agreement)—All evolutionary processes, including natural selection, genetic drift, mutation, migration and non-random mating, are important for understanding traits and disease. Many panelists made comments about the importance of recognizing the contributions of all four processes in order to avoid the error of considering only natural selection. Understanding evolution in depth is fundamental to evolutionary medicine, and this principle, while written to be general, captures how an understanding of all evolutionary processes is central to evolutionary medicine.

*Reproductive success* (96.6% agreement)—Natural selection maximizes reproductive success, sometimes at the expense of health and longevity. Initial survey responses included both general comments about the process of natural selection, as well as comments that specifically emphasized that natural selection selects for reproductive fitness, which can occur at the expense of health and longevity. While we initially considered these over-lapping principals (an understanding of natural selection free of misconceptions should include knowing that reproductive success can be at the expense of health and longevity), panelist comments and ratings indicated that a separate focus on reproductive success is an important and distinct core principle.

*Sexual selection* (80% agreement)—Sexual selection shapes traits that result in different health risks between sexes. Like the principle for reproductive success, sexual selection can be considered nested within a general understanding of natural selection. However, most panelists recommended including sexual selection as an important separate core principle. Understanding how sexual selection shapes differences in male and female physiology and behavior is important for understanding differences in health risks.

*Constraints on natural selection* (90% agreement)—Several constraints inhibit the capacity of natural selection to shape traits that are hypothetically optimal for health. They include path dependence, the inevitability of mutations, trade-offs such those seen in antagonistic pleiotropy and others. This is a principle with large scope, and large ideas in evolutionary medicine nested within it. The vague wording of this principle led to some concerns about its importance, but most panelists saw this idea as an important idea for the field.

*Trade-offs* (96.5% agreement)—Evolutionary changes in one trait that improve fitness can be linked to changes in other traits that decrease fitness. The role of evolutionary trade-offs in explaining disease vulnerability is a central and important core principle for evolutionary medicine. The principle is intimately tied to Life History Theory, and has been a major and influential idea in Evolutionary Medicine and beyond [10]. Indeed, some panelists felt that LHT was a nested principle that could be understood through trade-offs, while others saw this relationship in the inverse (trade-offs as a subset of LHT). While this principle achieved high agreement in its current form, it could be somewhat misleading by implying that trade-offs must include two traits. An alternative wording could be ‘Evolutionary changes in a trait that improve fitness can often also decrease fitness’. For instance, lower levels of gastric acid reduce ulcers at the cost of increased risk of infection.

*Life history theory* (100% agreement)—Life history traits, such as age at first reproduction, reproductive lifespan and rate of senescence, are shaped by evolution, and have implications for health and disease. The evolution of life history traits is intricately tied to many aspects of health. Understanding the evolutionary origins of human life history traits such as altriciality, short interbirth intervals and prolonged maturation time are critical for understanding life-stages and health outcomes. Initially, because of the close tie to trade-offs, LHT was listed as potentially over-lapping with trade-offs. However, responses in subsequent surveys indicated a consensus among panelists that LHT is important and unique enough to be listed as a distinct core principle.

*Multiple levels of selection* (86% agreement)—Vulnerabilities to disease can result when selection has opposing effects at different levels (e.g. genetic elements, cells, organisms, kin and other levels). Responses to the initial survey suggested listing somatic selection in cancer, genetic conflicts and mentions of group selection as core principles. While these concepts differ in their implications for health and disease, they share a larger focus on thinking about selective dynamics at levels other than the individual. That is, natural selection can act on replicating entities at different levels, and when the selection forces differ between these levels, conflict can occur. Thus, understanding cancer through an evolutionary lens requires considering the how selective dynamics at the cellular level interact with those at the individual level. Similar reasoning is needed to understand the evolutionary dynamics of genetic element replication at a cost to the cell.

*Phylogeny* (88.5% agreement)—Tracing phylogenetic relationships for species, populations, traits or pathogens can provide insights into health and disease. While neglected early on in evolutionary medicine, tracing phylogenetic relationships is a major area of evolutionary research that is becoming increasingly important for evolutionary medicine. While phylogenies often focus on the relation between species, comments from panelists indicated that phylogenies of populations, different molecules or traits and pathogens, are all useful for medical research. This core principle encompasses the importance of understanding the relatedness between any replicating entities.

*Coevolution* (96.5% agreement)—Coevolution among species can influence health and disease (e.g. evolutionary arms races and mutualistic relationships such as those seen in the microbiome). Understanding many human diseases requires appreciating the coevolution between pathogens and defenses against those pathogens. Indeed, most anti-biotics are produced by bacteria as a result of coevolutionary competitions with viruses and other bacteria. Notably, coevolution is also an important consideration for the emerging field investigating the roles of microbiomes in health.

*Plasticity* (96.5% agreement)—Environmental factors can shift developmental trajectories in ways that influence health and the plasticity of these trajectories can be the product of evolved adaptive mechanisms. Plasticity is a general capacity of living organisms—phenotypes shift in the course of development or over shorter time frames as genes interact with varying environments. Plasticity is important to evolutionary medicine because selection shapes mechanisms that regulate plasticity that can influence disease risks. Especially important for medicine are mechanisms that shift development in response to environmental cues detected during developmental windows.

*Defenses* (93% agreement)—Many signs and symptoms of disease (e.g. fever) are useful defenses, which can be pathological if dysregulated. Evolved defenses as a concept has a more narrow focus than some of the other principles, but it is centrally important to how evolution can inform medicine. Understanding signs and symptoms of disease as protective responses has implications for treatment. How selection shapes systems that regulate defense expression (the Smoke Detector Principle) was considered as a separate principle but was incorporated into this larger category.

*Mismatch* (93% agreement)—Disease risks can be altered for organisms living in environments that differ from those in which their ancestors evolved. Many panelists mentioned the idea of evolutionary mismatch in the first survey. Comments throughout the Delphi study necessitated edits to ensure that this principle captured the various ways mismatch can occur (e.g. moving to a new environment, a past environment changing rapidly, etc.). It is also important to avoid the incorrect assumption that humans are adapted to a single environment, and to recognize that mismatch may result from migration between stable environments [35].

*Cultural practices* (100% agreement)—Cultural practices can influence the evolution of humans and other species (including pathogens), in ways that can affect health and disease (e.g. anti-biotic use, birth practices, diet, etc.). Understanding any aspect of human traits requires considering the importance of culture and cultural practices. While a general consideration of human culture is critical to understanding human evolution [50], it is also important in many aspects of human health. This importance includes the evolutionary impacts of medical practices such as anti-biotic use, chemo-therapy regimens and caesarean sections. This principle can incorporate the importance of many behaviors and traits not-attributable to genetics, but possibly involving cultural practices.

### Principles not meeting consensus

Many principles nominated by the panelists either did not achieve 80% consensus regarding their importance to evolutionary medicine, and one (natural selection) was above this threshold but a similar core principle (Reproductive success) was at a higher consensus. While the lower ratings of these principles resulted in their exclusion from the final list of core principles, we present them here (Table 3) for the interest and potential use of readers. Indeed, while these principles did not make the final list, many of them may still be of interest to instructors in creating learning goals in courses focused on related topics. Notably, some of the principles in this list could be considered more uniquely relevant to evolutionary medicine than other broader core principles. It is noteworthy that many of these specialized principles can be derived from the more generalized core principles, so it is possible that they were considered too specific to be a core principle. For example, combining an understanding of evolved defenses and trade-offs allows one to grasp the smoke detector principle’s utility in understanding anxiety and other defense responses. Likewise, Developmental Origins Of Health And Disease is a specific application of phenotypic plasticity.

**Table 3.** Principles suggested in the initial survey that fewer than 80% of panelists rated as core principles of Evolutionary Medicine

Suggested principles that didn't reach 80% consensus		% Agreement with statement as a core principle
Natural selection	Competition between variants for representation in future generations has shaped all aspects of our biology, and results in adaptations.	86.5%
Ethics	Applying evolution to studying and practicing human biology and medicine requires important ethical considerations given historical and current misappropriations.	70.3%
Variation	Variation is an intrinsic property of biological and cultural systems that can be inconsistent with tendencies to view all items in a category as identical (e.g. cells in a tumor, genotypes in a population)	67.8%
Developmental Origins of Adult Health and Disease (DOHaD)	Across ontogeny there are sensitive windows of organization where environment particularly influence that organization.	67.6%
Genomics	Genomes consist of protein coding and non-coding regions, both of which are important aspects to understand.	62.2%
Smoke detector principle	It is less costly for individuals to raise false alarms than it is to miss a signal, which results in sensitive systems.	59.5%
Epidemiological transition	A patterned change in public health and sanitation results in changed patterns of disease prevalence.	56.8%
Genomic conflict	Misalignment in evolutionary interests between genomic elements creates conflict.	54.1%
Hologenome theory of evolution	All complex organisms have a microbiome and selection operates at the level of the holobiont.	51.4%
Kin selection	Individuals can increase their fitness through increasing the reproductive success of their kin.	51.4%
Somatic selection	Single cell lines proliferate at the expense of other cells (i.e. cancer and clonal selection).	48.7%
Old friend's hypothesis	Changes in the communities of symbiotic microbes living with humans impacts human health.	45.9%
Group/cultural group selection	Selection at the group or cultural group level produces unique behavioral traits.	27%

It became apparent early on in the Delphi process that the network of core principles contained hierarchies; some specified ideas listed by panelists could be understood through an understanding of other broader ideas. These broader ideas tended to be derived from general evolutionary biology or medical sciences. Comments from panelists throughout the Delphi process illustrated the blurred line between evolutionary medicine and general evolutionary biology or medical sciences. This ongoing contention was often in the way of consensus among panelists, and illuminates a discussion as to whether ‘Evolutionary medicine’ is a separate field of study, or if it is better conceptualized as applying the principles of evolutionary biology in medicine. The result that the identified core principles trended toward broader ideas lends credence to evolutionary medicine being a subfield of evolutionary biology, with critical inputs from other disciplines. As an emergent and growing field, this may change over-time.

## DISCUSSION

The current study was designed to identify ‘the core principles of evolutionary medicine’, with the expectation that they will be useful to guide curricular development. These core principles could be especially useful for creating learning objectives for courses in evolutionary medicine in a way that aligns with national recommendations for teaching big ideas, and not isolated facts [1]. The principles elicited came from the evolutionary medicine community, and they represent ideas central to the field with broad applications. With this in mind, the core principles elicited here should not be interpreted as prescriptive, and should instead be thought of as a recipe for the development of learning objectives that encourages users to add or subtract core principles to their own needs. Similar to other efforts to present a set of core principles [45,51,52], the goal is to provide a resource for instructors, but not meant to constrain them. Further, as we highlighted, disagreements among panelists about some of the principles highlight that core ideas in this field will continue to evolve over-time.

Although there have been previous efforts to delineate the important concepts in evolutionary medicine, our efforts represent the first systematic study to do so with the involvement of over 50 individuals. The list of core principles is generally consistent with those emphasized in previous articles based on less systematic methods [25,29,31,53]. While these articles did not necessarily aim to define core principles with the same definition adopted here, or have a focus on being exhaustive, it is nonetheless instructive to examine the over-lap between list here and principles discussed in previous work. By doing so, we get some idea of the reliability of the results. Table 4 lists principles, learning goals, and suggested biomedical examples of evolutionary concepts as worded in previous articles. We denote in the table how these ideas over-lap with the core principles elicited here. While many of these are directly congruent with the core principles, others are more specific or even common misconceptions related to a core principle. We would argue that by the nature of our study design, the community of evolutionary medicine can have more confidence that our core principles are a consensus view. Thus, we hope that it can spur greater emphasis on these topics in evolutionary medicine courses so that there can be greater commonalities between evolutionary medicine courses taught by different instructors at different institutions.

**Table 4.** Principles, learning goals, and concepts as described in previous articles about evolutionary medicine

Source	Concept	CCP	SMCP	CWSP
Nesse et al. [29]	*Demonstrate an understanding of how natural selection shapes traits in organisms.*	*X*		
	*Describe the differences between proximate and evolutionary explanations, and the two subtypes under each.*	*X*		
	*Describe the mathematical formulations that describe the rate of change of an allele’s frequency under different strengths of selection, and the implications for hypotheses about the role of selection in accounting for differences among human populations.*		*X*	
	*Explain how the comparative method and other strategies can be used to test evolutionary explanations.*	*X*		
	*Be able to describe the role of tradeoffs in traits shaped by natural selection.*	*X*		
	*Understand the core principles of behavioral ecology.*			
	*Describe phenomena explained by kin selection and inclusive fitness more generally.*			*X*
	*Understand sexual selection, and how it can shape sex differences.*	*X*		
Gluckman et al. [53]	*We are now living in novel environments compared to those in which we evolved.*	*X*		
	*Selection acts on fitness, not health or longevity.*	*X*		
	*Our evolutionary history does not cause disease, but rather impacts on our risk of disease in particular environments.*		*X*	
Antolin et al. [31]	*Genetic variation is the material for evolutionary processes.*			*X*
	*Common descent is a result of evolution.*	*X*		
	*Adaptations within populations arise through the process of natural selection in particular environments.*	*X*		
	*Phenotypic expression of traits often varies across a range of environmental conditions and provides a predictive framework for potential responses to selection.*	*X*		
	*Life span evolves in the context of trade-offs between traits that influence fitness early versus later in life.*	*X*		
	*Evolutionary rate is dependent on generation times.*		*X*	
	*Humans have coevolved with a variety of commensal and pathogenic organisms*	*X*		
Graves et al. [25]	*Adaptation/adaptive.*	*X*		
	*Hygiene hypothesis.*		*X*	
	*Life history theory.*	*X*		
	*Microbiome.*	*X*		
	*Mismatch.*	*X*		
	*Natural selection*	*X*		
	*Race (biological and socially defined).*			*X*
	*Trade-offs.*	*X*		

CCP, Congruent with core principle; SMCP, Specific manifestation of a core principle; CWSP, Congruent with a sub principle.

Defined partially by their explanatory breadth and importance to the field, core principles also provide a framework that can organize research. The framework of core principles provided here can help clarify connections between ongoing research that may be based on larger ideas, and not on topics or methodology. Organizing research by large ideas is not novel; conference sessions have been organized on ideas such as life history theory and trade-offs. However, making the network of core principles more explicit can catalyze further connections between research that applies a shared principle without sharing topical focus or methods, and could expedite new and exciting research avenues.

## CONCLUSION

We hope that instructors designing new courses or revising current courses in evolutionary medicine consider these larger principles when designing learning goals for students. Instructors are encouraged to incorporate these principles in ways that incorporates their own experience and expertise, and the unique goals of their own curriculum and institutions. Disciplinary core principles help align classroom contents to the most pertinent material, are teachable at variable depths, and have high connectivity to other ideas and areas of content. For these reasons, core principles can provide crucial foundations for thinking about learning goals in evolutionary medicine curricula. Ideally, these learning goals will be developed toward higher-level learning goals as described by Bloom’s taxonomy [25,30], an important step toward improving the reach and quality of evolutionary medicine education.

Evolutionary medicine is a young field that is growing fast along with many new courses in university curricula, new Centers and Institutes, and a thriving international society (evolutionarymedicine.org). Healthy growth of this field will be supported by effective pedagogy that starts with decisions about which principles are most important for students and professionals to understand, and focuses curricula on those principles. Core principles provide a scaffolding to organize a growing array of facts and concepts. This organization is of great use in educational contexts, and may even help speed learning in medical curricula by clarifying the connections among thousands of otherwise unrelated facts. The validated list of core principles in evolutionary medicine presented here provides a starting point for teachers, students and current and future researchers.

Box 1. The three definitions of ‘Core Principle’ provided to the panelists in each survey

*From Niemi and Phelan (2008):*

 *‘…organized around central concepts or principles, or “big ideas.” The nature of these concepts differs from domain to domain, but in general they are abstract principles that can be used to organize broad areas of knowledge and make inferences in the domain, as well as determining strategies for solving a wide range of problems’.*

*From Wiggins and McTighe (2005):*

 *‘By definition, big ideas are important and enduring. Big ideas are transferable beyond the scope of a particular unit…Big ideas are the building material of understanding. They can be thought of as the meaningful patterns that enable one to connect the dots of otherwise fragmented knowledge’.*

*From Shouse et al. (2007):*

 *‘Each [big idea] is well tested, validated, and absolutely central to the discipline. Each integrates many different findings and has exceptionally broad explanatory scope. Each is the source of coherence for many key concepts, principles, and even other theories in the discipline’.*

## Supplementary Material

Supplementary Data
