# Supplementary material for: Core principles of evolutionary medicine: A Delphi study
Source: Evol Med Public Health. 2017 Dec 26;2018(1):13–23. doi: 10.1093/emph/eox025 (PMC5822696; doi:10.1093/emph/eox025)
Supplement: Supplementary Data [file eox025_supp.docx]

**Supplemental Materials**

**Panelist expertise:**

**
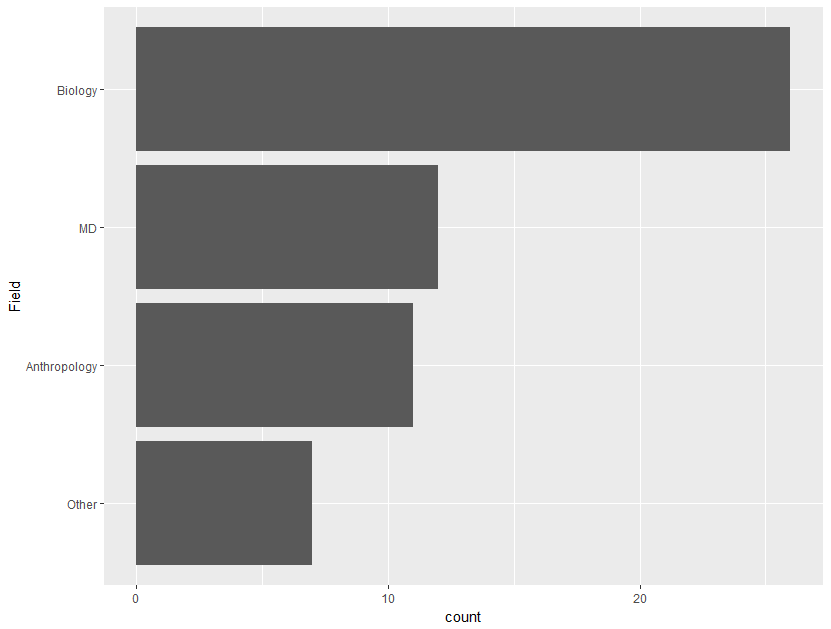
**

Panelists were classified by the researchers as primarily biologists, anthropologists, medical doctors, or ‘other’ kinds of researchers or professionals with expertise in evolutionary medicine.


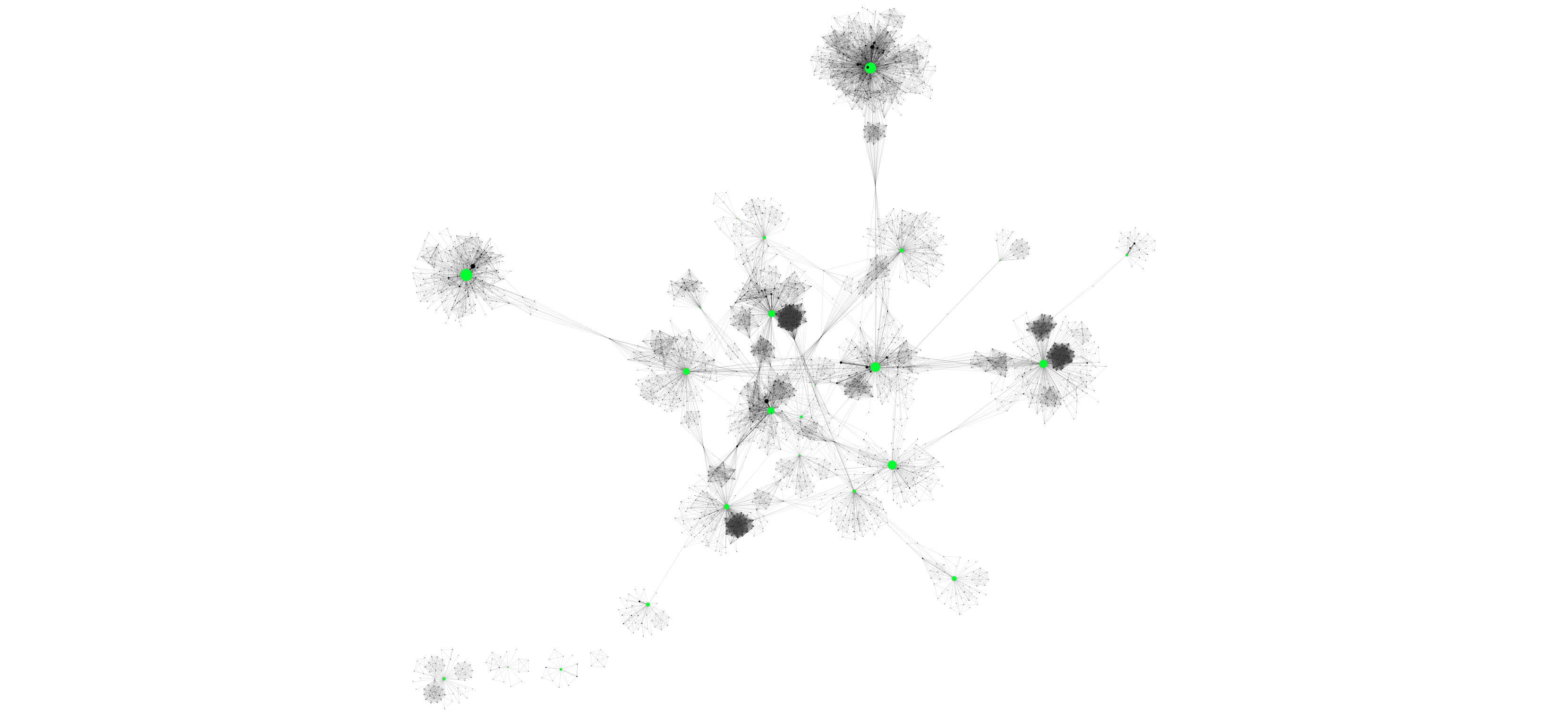


We created a co-authorship network representing the last 15 years of publications of panelists who responded to at least 3 of the 4 total surveys. Panelists are represented by green nodes, while black nodes represent co-authors who were not panelists. The size of nodes indicates the number of publications on PubMed over the past 15 years. The appearance of distinct network communities around each panelist indicates that these panelists tend to have distinct research interests and agreement is likely not based on extremely close authorship ties.

**Overview of surveys:**

*Survey 1*

Panelists were e-mailed a survey which explained the project and asked consent for participation. Following previous methods (McFarland and Michaels 2011), consenting participants were presented the following definitions of a “core principle”:

*From Duschl, Schweingruber and Shouse (2007):*

*“Each [big idea] is well tested, validated, and absolutely central to the discipline. Each integrates many different findings and has exceptionally broad explanatory scope. Each is the source of coherence for many key concepts, principles, and even other theories in the discipline.”*

*From Niemi and Phelan (2008):*

*“…organized around central concepts or principles, or ‘big ideas.’ The nature of these concepts differs from domain to domain, but in general they are abstract principles that can be used to organize broad areas of knowledge and make inferences in the domain, as well as determining strategies for solving a wide range of problems.”*

*From Wiggins and McTighe (2005):*

*“By definition, big ideas are important and enduring. Big ideas are transferable beyond the scope of a particular unit…Big ideas are the building material of understanding. They can be thought of as the meaningful patterns that enable one to connect the dots of otherwise fragmented knowledge.”*

Panelists were asked to list as many core principles for the field of EvMed as they would like that follow the definitions above. Panelists were asked to elaborate on what each idea is and why it is important.

Twenty-seven panelists responded to the first survey. Two researchers independently analyzed responses. Each researcher created a collated list of principles that emerged from these data. For the most part, both researchers identified the same core principles, but some discussion was required to determine whether certain ideas in the panel responses were separate ideas from other ideas listed, or were better understood as ideas nested within a larger principle. For example: genetic conflict, somatic selection, and theories of group selection were all mentioned, and could either be considered as separate principles or as a larger principle encompassing “Selection at many levels.” After a larger discussion, the research team opted to present “sub-principles” as part of survey 2 to represent finer coarse ideas separate from the broader ones. While most responses included principles that conformed to the definitions given above, some responses were given as topics, such as “Cancer” or “Mental health.” The research team opted to not include these in the collated list. In total, 15 core principles and 12 sub-principles were identified from the first survey.

*Survey 2*

The 15 core principles were returned to the panel, who were asked to rate their agreement with the statement “*This principle is important for Evolutionary Medicine,”* on a five-point Likert scale from “Strongly disagree” to “Strongly agree,” and were also given space to leave comments regarding their response or the wording for the principle itself. The 12 sub-principles were also returned to the panelists with the same question asked, only with an added option “*This is not a distinct core principle.”* Space was again provided for comments.

Thirty-seven panelists responded to this second survey. Responses in the comment space indicated that many panelists were concerned with the wording of principles. Two researchers independently editing the wording of principles based on the comments of panelist responses. These researchers met to compare updated wordings of these principles, and merge them into new versions that addressed common panelist concerns. The full team of researchers met to discuss the updated principles and further refine them based on panelist comments.

Likert ratings regarding the importance of each principle were used to assess whether these 27 principles should be kept as core principles for the next survey, or removed from future consideration. In total, two principles that were considered core principles at the beginning of this second survey were removed from the list, while two sub-principles were promoted to the level of core principle. The rest of the ten sub-principles were removed from further consideration throughout the Delphi process.


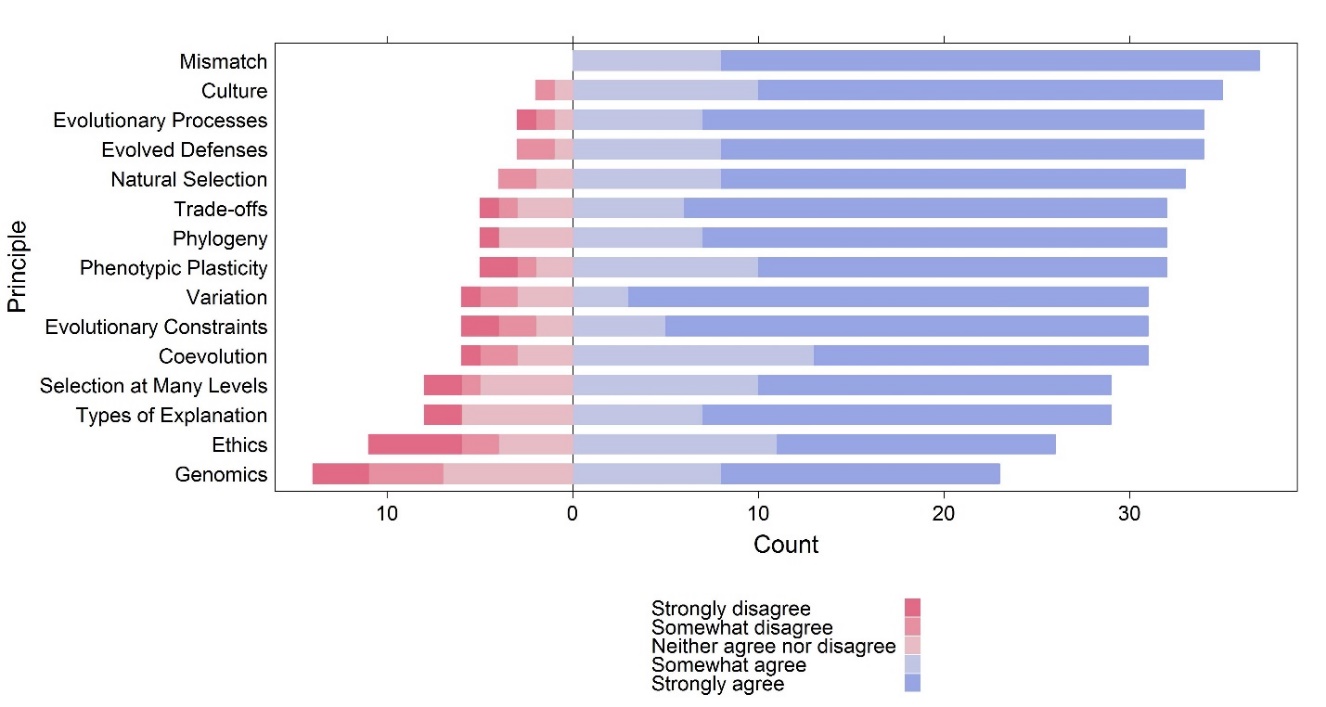


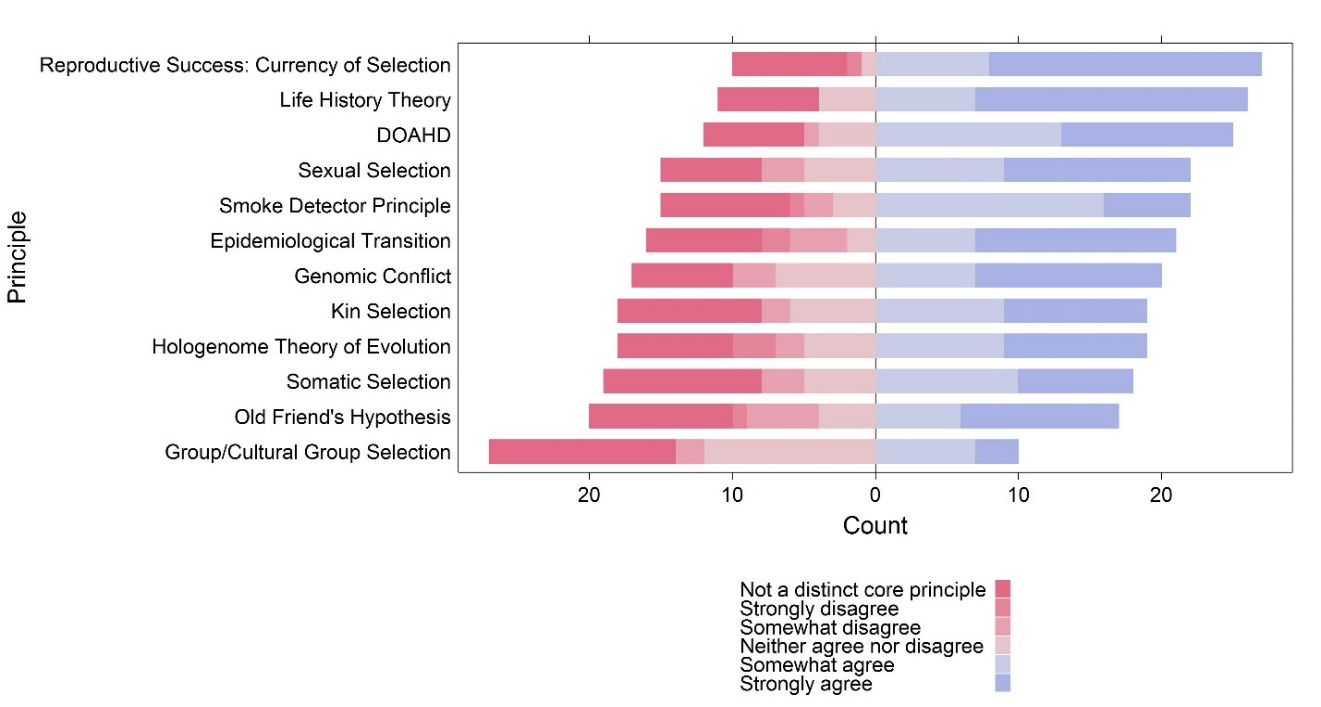


*Survey 3:*

The 15 reworded core principles from the second survey were returned to the panelists for a third survey. Panelists were asked to rate their agreement with the statement “*This principle is important for Evolutionary Medicine,”* on a 4 point Likert scale and to either agree or disagree with the statement *“This statement is scientifically accurate.”* Comment boxes were provided for panelists to elaborate on their responses. Along with the survey, summary data of results from round two, a list of changes made to the principles, and a copy of the panelist’s round two responses (if they responded) were returned to all panelists.

Thirty panelists responded to the third survey. Responses indicated issues with the wording of 12 of the principles that necessitated editing to the wording of the principles. For example, one word in the core principle “Phylogeny” was found to be confusing by many panelists, so the wording was changed to remove this word. Three members of the research team went through these comments and edited these principles based on these comments. The other three principles were kept as is.


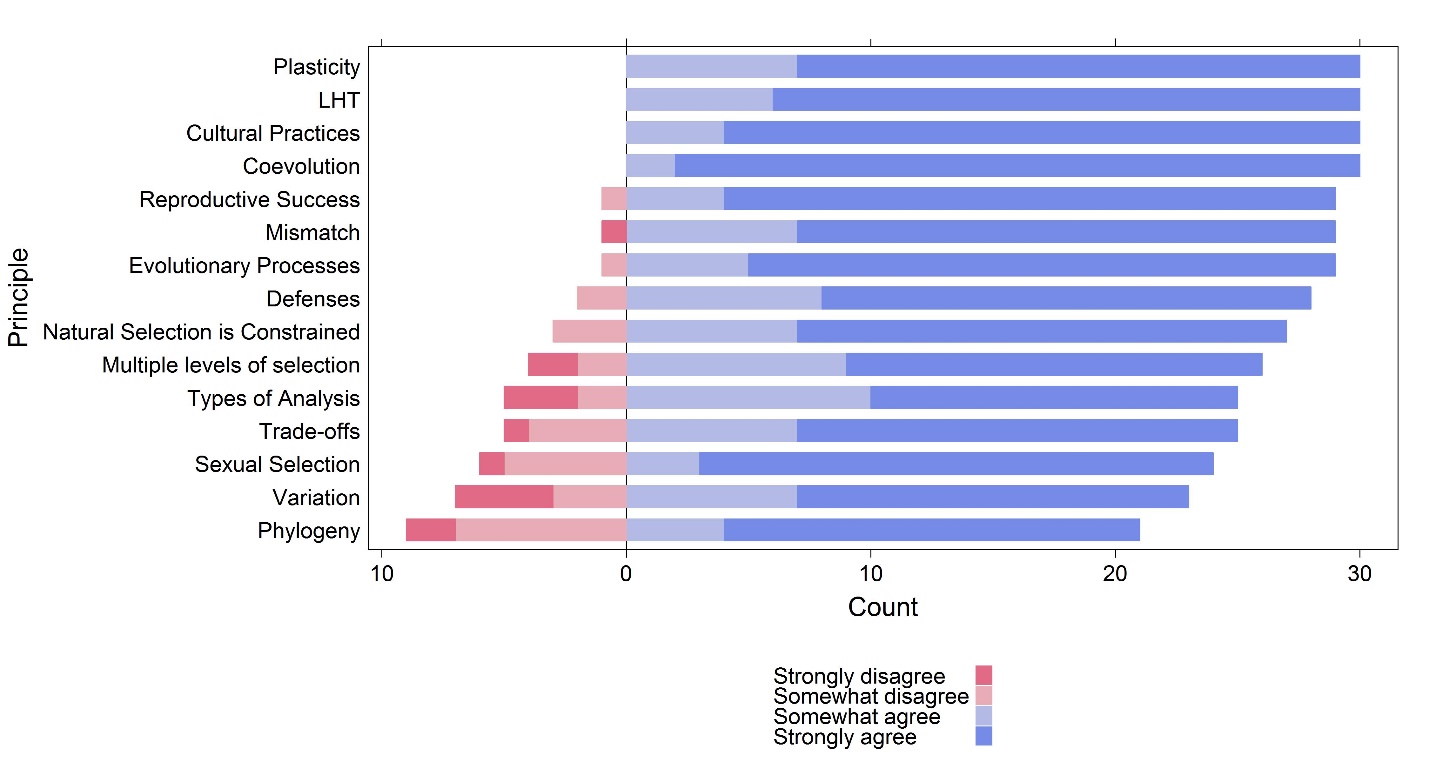


*Survey 4:*

A fourth and final survey was sent out with the 12 core principles edited after survey 3. Panelists were asked to rate their agreement with the statement “*This principle is important for Evolutionary Medicine,”* on a 4 point Likert scale. Panelists were also asked to agree or disagree with the statement *“This statement is scientifically accurate.”* Comment boxes were provided for panelists to elaborate on their responses.

Twenty-eight panelists responded to this survey. Consensus on the importance of each principle was reached for all but two principles (Variation and Natural Selection is Constrained). Based on the ratings and comments from the panel, decisions were made to: 1) revert to the wording of “Natural selection is constrained” used in the third survey, and 2) drop “Variation” from the core principles list.


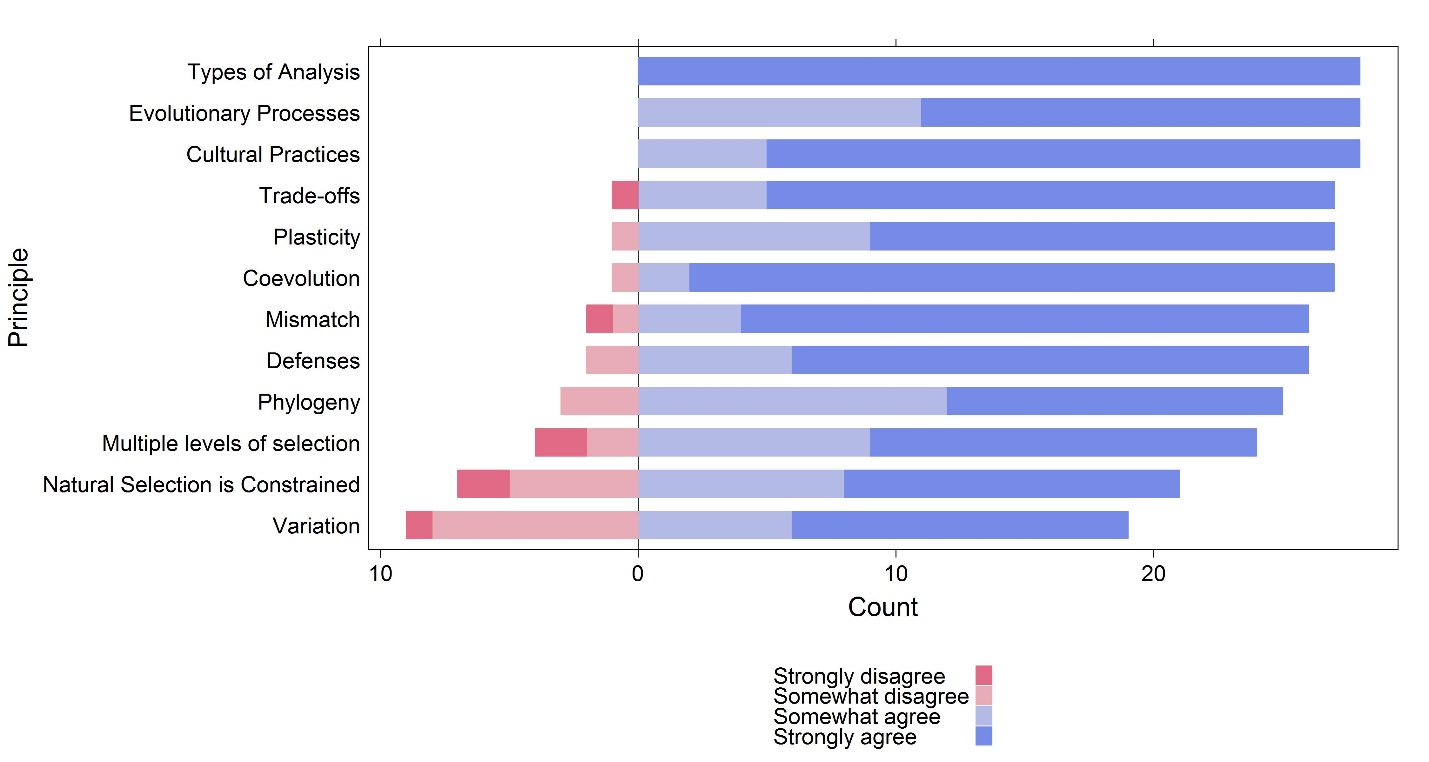


**Works cited:**

Michael J, McFarland J. The core principles (“big ideas”) of physiology: results of faculty surveys. *Advances in physiology education* 2011;**35**(4):336-341.

Shouse AW, Schweingruber HA, Duschl RA. *Taking science to school: Learning and teaching science in grades K-8*: National Academies Press, 2007.

Niemi DN, Phelan J; Eliciting Big Ideas in Biology. *Conceptual Assessment in Biology II Conference*. Asilomar, CA, 2008.

Wiggins GP, McTighe J. *Understanding by design*: Ascd, 2005.
